# Down-regulation of cell membrane localized NTCP expression in proliferating hepatocytes prevents hepatitis B virus infection

**DOI:** 10.1080/22221751.2019.1625728

**Published:** 2019-06-09

**Authors:** Ying Yan, Lena Allweiss, Danli Yang, Jingting Kang, Jianwen Wang, Xiangjun Qian, Ting Zhang, Hui Liu, Lu Wang, Shuhong Liu, Jianhua Sui, Xiangmei Chen, Maura Dandri, Jingmin Zhao, Fengmin Lu

**Affiliations:** a State Key Laboratory of Natural and Biomimetic Drugs, Department of Microbiology and Infectious Disease Center, School of Basic Medical Sciences, Peking University Health Science Center, Beijing, People’s Republic of China; b Department of Medicine, Center for Internal Medicine, University Medical Center Hamburg-Eppendorf, Hamburg, Germany; c Institute of Basic Medical Sciences Chinese Academy of Medical Sciences, School of Basic Medicine Peking Union Medical College, Beijing, People’s Republic of China; d Department of Pathology and Hepatology, The 5th Medical Centre, Chinese PLA General Hospital, Beijing, People’s Republic of China; e Biologics Research Center, National Institute of Biological Sciences, Beijing, People’s Republic of China; f German Center for Infection Research (DZIF), Hamburg-Lübeck-Borstel-Riems Partner Site, Hamburg, Germany

**Keywords:** Sodium taurocholate cotransporting polypeptide (NTCP), hepatocyte proliferation, hepatitis B virus (HBV) infection, chronic hepatitis B (CHB), antiviral therapy, p53, S-phase kinase-associated protein 2 (SKP2), cyclin D1

## Abstract

Hepatocyte proliferation could result in the loss of covalently closed circular DNA (cccDNA) and the emergence of cccDNA-cleared nascent hepatocytes, which appear refractory to hepatitis B virus (HBV) reinfection with unknown mechanism(s). Sodium taurocholate cotransporting polypeptide (NTCP) is the functional receptor for HBV entry. In this study, down-regulation of cell membrane localized NTCP expression in proliferating hepatocytes was found to prevent HBV infection in HepG2-NTCP-tet cells and in liver-humanized mice. In patients, lower NTCP protein expression was correlated well with higher levels of hepatocyte proliferation and less HBsAg expression in HBV-related focal nodular hyperplasia (FNH) tissues. Clinically, significantly lower NTCP protein expression was correlated with more active hepatocyte proliferation in CHB patients with severe active necroinflammation and better antiviral treatment outcome. Mechanistically, the activation of cell cycle regulatory genes p53, S-phase kinase-associated protein 2 (SKP2) and cyclin D1 during cell proliferation, as well as proliferative and inflammatory cytokine Interleukin-6 (IL-6) could transcriptionally down-regulate NTCP expression. From these aspects, we conclude that within the milieu of hepatocyte proliferation, down-regulation of cell membrane localized NTCP expression level renders nascent hepatocytes resistant to HBV reinfection. This may accelerate virus clearance during immune-mediated cell death and compensatory proliferation of survival hepatocytes.

## Introduction

Hepatitis B virus (HBV) infection is the major cause of chronic hepatitis B (CHB), cirrhosis, and hepatocellular carcinoma (HCC) worldwide. Although prophylactic HBV vaccines have been available for decades, the overall number of chronic infections remains high with >240 million worldwide [1]. Chronic HBV infection is still a serious global public health problem [2].

HBV infection in more than 95% of adults is a self-limited, transient liver disease, while more than 90% of neonates exposed to HBV become chronically infected [3]. During HBV infection, immune-mediated cytolytic processes destroy infected cells and induce proliferation of neighbouring HBV-infected hepatocytes to compensate for cell loss. Since it has been demonstrated that HBV infection can spread to the entire hepatocyte population in the acute phase and in the tolerant phase of chronically infected individuals, it is reasonable to postulate that the uninfected hepatocytes present in the recovered liver should be derived from previously infected hepatocytes [4,5]. Yet, how these cells clear the virus remains elusive.

The covalently closed circular DNA (cccDNA), originated from the relaxed circular DNA (rcDNA), carries the full genetic information required for HBV replication, which is the sole source of persistent HBV replication and establishment of persistent infection [5–7]. Using integrated viral DNA as a genetic marker of the infected cell population, Summers et al. indicated that during the clearance of HBV infection, proliferation is equivalent to a minimum of 0.7 complete turnovers of the hepatocyte population [5]. cccDNA in the quiescent hepatocyte nucleus appears to have a long half-life and long-term stability. However, since it exists as an extrachromosomal plasmid-like structure in the nucleus lacking centromeres, the cccDNA is very likely to distribute in an unequal manner between daughter cells when HBV-infected hepatocytes divide, eventually leading to the formation of cccDNA-free cells [8]. Many studies involving patient liver biopsies and animal experiments have shown a negative correlation between hepatocyte proliferation and cccDNA load [8,9]. Moreover, using the HBV-infected humanized mouse model, a recent study proved for the first time that HBV-infected human hepatocyte division triggers substantial cccDNA dilution and loss [10].

Besides cccDNA dilution and loss during proliferation, it has been noticed for a long time that the nascent hepatocytes are protected from reinfection or de novo infection [9,11–13], which may further help virus clearance. However, how this occurs is paramount but unsolved. Although inflammatory factors and the immune of host cells were speculated to play roles in it, there were no direct supportive experimental evidences [5,14].

Sodium taurocholate cotransporting polypeptide (NTCP) is the most important conjugated bile acid transporter located on the basolateral membrane of hepatocytes, which can transport about 80% bile acids from blood into liver in a sodium-dependent manner [15]. NTCP has also been proved to be the functional receptor for HBV entry and its normal expression is closely related to HBV infection [16–18]. It has been reported that NTCP expression was dramatically decreased in rats after 90% hepatectomy, and returned to normal immediately after liver reconstruction was completed [19]. In addition, our previous study reported that NTCP was down-regulated in HCC tissues and was up-regulated when HCC cell lines were arrested in the G0/G1 phase [20]. Combining these studies, it seems that the down-regulation of NTCP in proliferating hepatocytes may be the reason that the regenerative hepatocytes are protected from reinfection or de novo infection. Unfortunately, there are only very few studies exploring in depth the mechanisms of NTCP down-regulation in proliferating hepatocytes.

In this study, we aimed to clarify whether the lower expression of cell membrane localized NTCP could influence HBV de novo infection or reinfection, to examine the possible clinical implications of this phenomenon, and to explore the molecular mechanisms relevant to NTCP down-regulation in proliferating hepatocytes. Through these aspects, we hope to provide new clues and insights for CHB treatment and cure.

## Materials and methods

### Animals

Male C57BL/6 mice (6–8 weeks old) were purchased from Department of Laboratory Animal Science of Peking University Health Science Center. All experimental researches on animals followed internationally recognized guidelines.

### Cell cycle assay

Cells were harvested in 70% ethanol at −20°C overnight. The fixed cells were resuspended with 100 µL 50 µg/mL RNase A (Nanjing KeyGen Biotech Co., Ltd.), and incubated at 37°C for 30 min. Then 100 µL propidium iodine (PI) solution (Nanjing KeyGen Biotech Co. Ltd.) was added and the ﬂuorescence of the PI labelled cells was measured using a ﬂow cytometer (FACS Calibur, BD Biosciences, San Jose, CA, USA).

### Human HCC cell lines and cell transfection

Human HCC cell lines HepG2, Hep3B, Huh-7, and SNU449 were purchased from the American Type Culture Collection (Manassas, VA, USA). Human HCC cell line SMMC7721 was purchased from Cell Resources Center of Peking Union Medical College (Beijing, P.R. China), and the origin of SMMC7721 has been proved with STR, hepatic gene expression and specific gene mutations. HepG2-NTCP-tet cell line was kindly gifted by Professor Ningshao Xia (Xiamen University, Xiamen, China). The NTCP expression of this cell line was under control of a CMV-Tet-on expression system. HepG2-NTCP cell line constantly expressing flag-tagged NTCP under control of the CMV promoter was constructed and the expression of exogenous NTCP was demonstrated before [20]. Cells were cultured in either Dulbecco’s modified Eagle’s medium (DMEM) or Roswell Park Memorial Institute (RPMI)-1640 supplemented with 10% fetal bovine serum (Gibco, Life Technologies, Carlsbad, CA, USA) in a humidified incubator maintaining 37°C and 5% CO_2_. To induce cell cycle arrest, cells were cultured in hepatocyte culture medium (HCM) (Lonza, Walkersville, MD, USA), a serum-free medium containing 1‰ hydrocortisone and other reagents, and 2% dimethyl sulfoxide (DMSO) was added in addition to further block the cell cycle progression. The HCM mentioned below was all added with 2% DMSO. Cells transfections with plasmids were conducted using Lipofectamine 2000/3000 Transfection Reagent (Invitrogen, Carlsbad, CA, USA) according to the manufacturer's instructions.

### HBV infection

The supernatant of HepAD38 cells was concentrated using 6×PEG8000 buffer (48% PEG8000 and 200 mM NaCl). The HepG2-NTCP-tet cells were cultured with 4μg/mL doxycycline (DOX) for 4 days to induce NTCP expression, and seeded in a 24-well plate and maintained in DMEM or HCM for 24 h. Cells were then incubated with different concentrations of HepAD38 cell supernatant in the presence of 4% PEG8000 and 2% DMSO for 24 h. HepG2-NTCP-tet cells were then washed with PBS for six times and maintained in DMEM with medium changed every 2 days.

### Quantification of HBsAg and HBeAg

The levels of HBsAg and HBeAg were measured by the diagnostic kit for the quantitative determination of HBsAg or HBeAg (time-resolved Immunofluorometric Assay) (PerkinElmer Inc., Suzhou, China) following the manufacturer's instructions.

### Immunofluorescence of NTCP in HepG2-NTCP-tet cells

For immunofluorescence, HepG2-NTCP-tet cells were fixed in 4% paraformaldehyde and then blocked with PBS containing 5% goat serum. Cells were incubated overnight with primary antibody NTCP (36C1M, 1 : 200) [21] at 4°C, followed by secondary antibodies Rhodamine (TRITC)-Conjugated Goat anti-Mouse IgG (Origene, ZF-0318, 1:50) for 1hr at room temperature. Finally, the samples were counter-stained with Hoechst (10 μg/mL) for 10 min, and photographed with confocal microscopy or fluorescence microscope.

### Immunofluorescence of NTCP in liver-humanized mice

Homozygous uPA/SCID/beige mice (shortly termed USB) were housed and maintained under specific pathogen-free conditions according to institutional guidelines under authorized protocols [10]. The generation of humanized mice and the establishment of a serial transplantation to induce hepatocyte proliferation were described previously [10]. Immunofluorescence on cryopreserved liver sections was performed as previously described [10] using the antibodies anti-NTCP (Sigma, St. Louis, MO, USA), anti-Ki67 (Dako Diagnostika, Glostrup, Denmark) and anti-SP100 (kindly provided by Hans Will); Anti-keratin 18 (Santa Cruz Biotechnology, Dallas, TX, USA) was used with anti-HBcAg antibodies (Dako Diagnostika, Glostrup, Denmark).

### Patient specimens

From January 2011 to March 2015, 68 patients in the 5th Medical Centre, Chinese PLA General Hospital who fulfilled the study criteria were enrolled. The inflammation activity stage of the enrolled CHB patients had been diagnosed by pathologic examination following the Practice Guidelines.

### Immunohistochemical double staining

5 μm thick liver tissues microarray sections were grilled at 80°C for 30 min, and then stained by using the Ventana NexES Staining System following the manufacturer's instructions. The antibodies used are anti-NTCP (P17-39, 1:4000); [20] anti-Ki67 (Origene, ZM-0166, 1:200); anti-HBsAg (Origene, ZM-0122, 1:50).

### Real-time reverse transcription (RT)-PCR

Real-time RT-PCR was performed as described previously [22]. Glyceraldehyde-3-Phosphate Dehydrogenase (GAPDH) was used as the reference gene to determine gene(s) expression. The primers used for real-time RT-PCR are listed in Supplemental Table 1.

### Western blot analysis

Western blot analysis was performed similarly as previously described [22]. Briefly, the lysed cell supernatant was run on a SDS-PAGE, and blotted with antibodies. The antibodies used in western blot are anti-flag-tag (MBL, M185-3L, 1:1000); anti-p53 (CST, 2524 T, 1:1000); anti-GAPDH (Abgent, AM1020b, 1:8000); anti-Beta-actin (Abgent, AW5206, 1:8000).

### Luciferase reporter assay

The conserved region of human NTCP promoter was amplified using PrimeSTAR HS DNA Polymerase (Takara, Dalian, China) and inserted into pGL3-Basic plasmid. p53 and E2F binding site mutations were amplified using the plasmid as template. Actin-renilla was used as the reference plasmid. All primers used are in Supplemental Table 2. Dual-luciferase reporter assays were carried out as described previously [20].

### Chromatin immunoprecipitation (ChIP) assay

The ChIP assay was performed as described previously [22]. The lysates of mouse liver tissues or SMMC7721 cells transfected with the p53 expression plasmid were incubated with rabbit anti-p53 antibody (Abcam, ab131442, 1:100) or immunoglobulin G from rabbit serum (Sigma, St. Louis, MO, USA). Huh-7 cells were transfected with the E2F1 expression plasmid. The lysates were incubated with rabbit anti-E2F1 antibody (Abcam, ab179445, 1:200) or immunoglobulin G from rabbit serum (Sigma, St. Louis, MO, USA). The primers used for validation the human NTCP promoter are as follows: NTCP promoter-sense: TGACAAGGGAGGAGTACAAGTAGCACCCAG; NTCP promoter-antisense: CCTCCTGTGAGGCAGTGGAAGACCACTCC. The primers used for validation of the mouse NTCP promoter are: NTCP-promoter-sense: TAGTGAAGCATGCTCAGCAGGGTAA; NTCP promoter-antisense: GACCCAGTGAAC ACCACCTC.

### Statistical analysis

Statistical analysis was performed with the SPSS18.0. Fisher's exact probability test was used to compare categorical variables and non-parametric Mann–Whitney U test was used for continuous variables. The data involved in CHB patients subjected to normal distribution, differences between two groups were analysed by 2-tailed Student t-test. *P* value < 0.05 (two-tailed) were considered to be statistically significant. *P < 0.05; **P < 0.01; ***P < 0.001; ns: not significant.

## Results

### Elevated cell membrane expression of NTCP in HepG2-NTCP-tet cells increases HBV infection susceptibility

**Figure 1.**
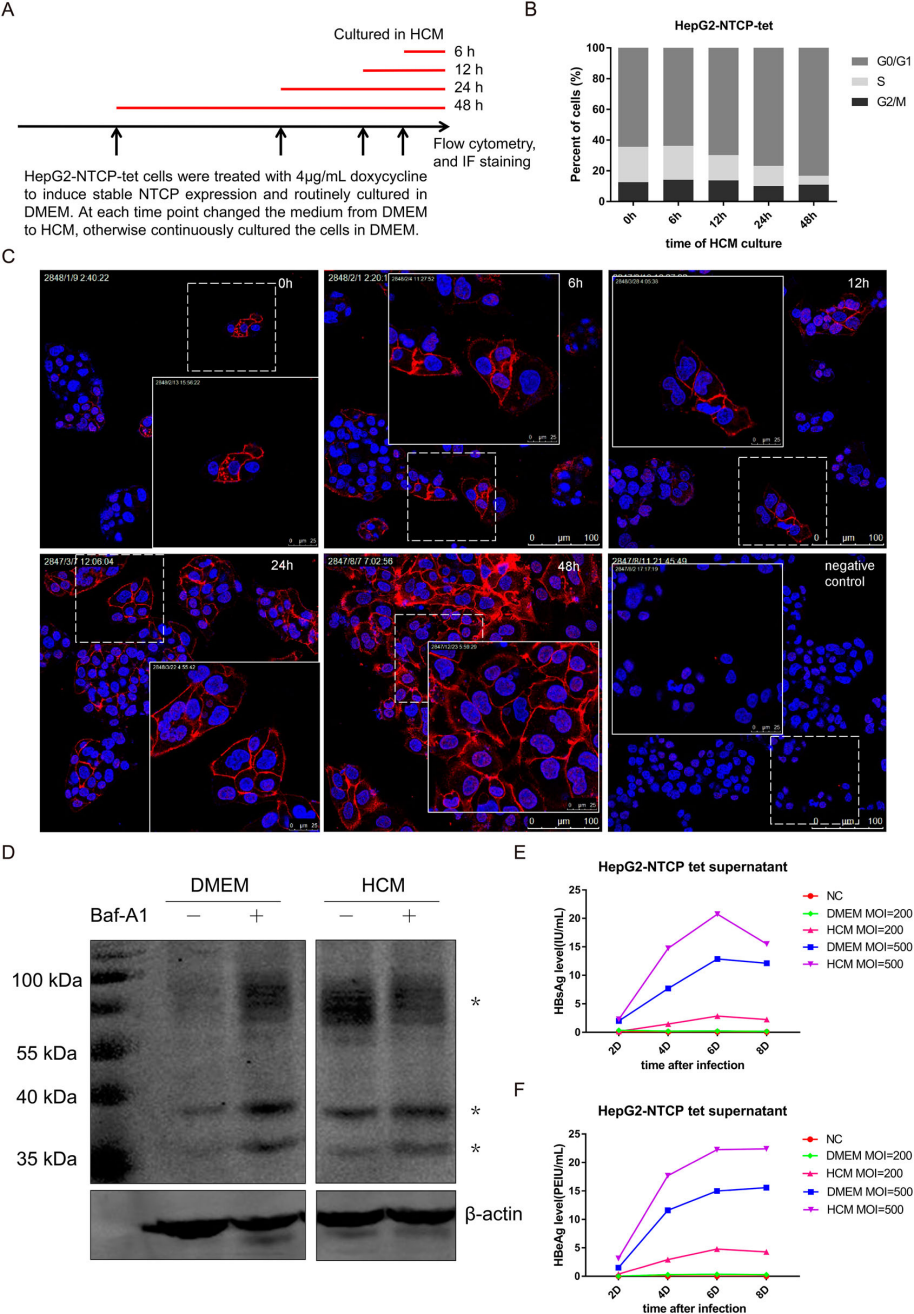
Elevated cell membrane expression of NTCP in HepG2-NTCP-tet cells increases HBV susceptibility. (A) The schematic diagram of treating HepG2-NTCP-tet cells in HCM medium for different time points. (B) Percentage of DOX-treated HepG2-NTCP-tet cells in each phase of the cell cycle (tested by flow cytometry cell cycle assays) at the different time points of HCM culture. (C) Immunofluorescent staining for NTCP of DOX-treated HepG2-NTCP-tet cells cultured in HCM for different times. HepG2-NTCP-tet cells without DOX treatment as negative control. (D) HepG2-NTCP cells were cultured in DMEM or HCM respectively for 24 h, and then treated with or without 10 nM Baf-A1 for another 24 h. The NTCP protein was tested using anti-flag-tag by western blot. “*” represents different bands of NTCP protein. (E, F) Changes of HBsAg and HBeAg in cell culture supernatant of HepG2-NTCP-tet cells at different time points after infected with the HBV particles concentrated from the HepAD38 cell culture supernatant. NC: negative control, standing for uninfected cells; DMEM MOI = 200 or DMEM MOI = 500: cells cultured in DMEM with the infection MOI of 200 or 500; HCM MOI = 200 or HCM MOI = 500: cells cultured in HCM with the infection MOI of 200 or 500.

To explore whether NTCP is down-regulated during hepatocyte proliferation, HepG2-NTCP-tet cells were routinely cultured in DMEM and treated with 4 μg/mL DOX for 4 days and all along afterwards to induce and maintain stable NTCP expression. After that, cells were treated with HCM for different time points, as indicated in Figure 1(A). The flow cytometry cell cycle assays showed that cells were progressively arrested in G0/G1 phase with the prolonged HCM culture time (Figure 1(B)). Meanwhile, the proportion of NTCP positive cells and the staining intensity of cell membrane localized NTCP were significantly increased (Figure 1(C)). The mRNA level of NTCP did not obviously change with the prolonged HCM culture time (Supplemental Fig 1), suggesting that hepatocyte proliferation is unlikely to regulate NTCP expression at the transcriptional level in this cell line. To further explore the mechanism relevant to the increase of NTCP protein level after cell cycle arrest, a HepG2 cell strain stably expressing ectopic flag-tagged NTCP under the control of CMV promoter was used. The cells were cultured either in DMEM or HCM medium, respectively. As shown in Figure 1(D), NTCP protein was detected as multiple bands due to glycosylation modification [23]. Compared to those cultured in DMEM, a relative higher expression of NTCP protein in cells cultured in HCM medium was observed, which was in concordant with the result demonstrated by immunofluorescent staining in HepG2-NTCP-tet cells (Figure 1(C)). Furthermore, after using Bafilomycin A1 (Baf-A1) to inhibit the lysosomal degradation of cellular protein, the NTCP protein level was found to increase largely in the group of DMEM culture. In contrast, no further increase of total NTCP protein level was observed in the cells cultured in HCM medium, after the same Baf-A1 treatment. This result showed that culturing cells in HCM medium could at least partially inhibit the degradation of NTCP protein by lysosomal degradation pathway, which suggested that the stabilization of NTCP protein could be a reason contributed to the upregulation of NTCP protein level when cells were arrested in G0/G1 phase.

Given that human NTCP is the major functional receptor of HBV, it seems reasonable to presume that the subcellular localization and the cell membrane level of human NTCP protein might influence HBV infection. To confirm this, DOX-treated HepG2-NTCP-tet cells were cultured in DMEM or HCM for 24 h, and were infected with the HBV particles concentrated from the HepAD38 cell culture supernatant. Compared with cells cultured in DMEM, cells cultured in HCM had higher HBsAg and HBeAg levels in the cell culture supernatant per multiplicity of infection (MOI) and per time point after infection (Figure 1(E, F)), indicating that low level of NTCP at the cell membrane is adverse to HBV infection, which may explain why proliferating hepatocytes are protected from reinfection or de novo infection.

### Proliferating human hepatocytes in liver-humanized mice are characterized with lower cell membrane localized NTCP expression and refractory to HBV infection

As previously reported [10], after transplantation of HBV-infected human hepatocytes into USB mice, spreading of HBV infection could not be observed during the early liver reconstitution process, despite the presence of low levels of circulating virions (10^5^ copies/mL HBV DNA in serum), whereas the virological markers began to rebound only after the reconstruction process was completed and hepatocyte proliferation had stopped.

**Figure 2.**
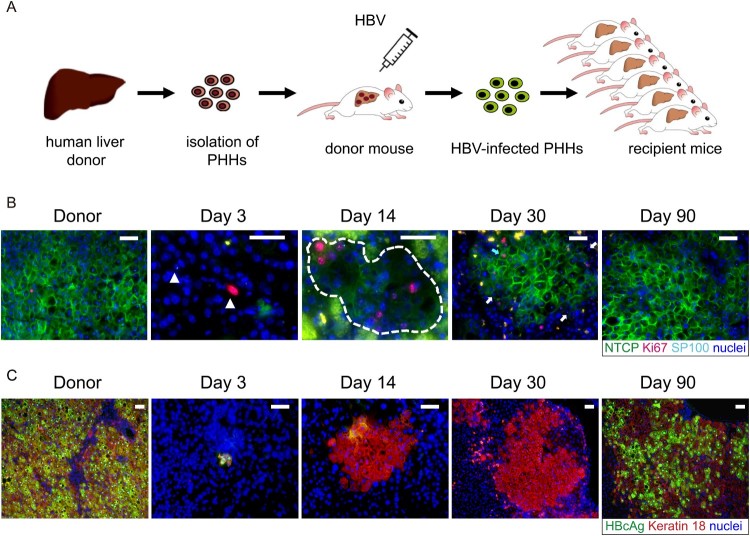
HBV reinfection resistance of proliferating hepatocytes seen in liver-humanized mouse model could be due to NTCP down-regulation. (A) Schematic diagram of the steps used to produce chronically HBV-infected USB mice (first transplantation) and to induce proliferation of HBV-infected human primary hepatocytes through a second transplantation. (B) Immunofluorescent staining of frozen sections for NTCP (green), Ki67(red), SP100 (light blue small dots in the nuclei) and nuclei (blue). SP100 was used to specifically recognize human hepatocytes in the mouse livers. The white triangles in picture of day 3 and broken white line in picture of day 14 depict human hepatocytes. The blue arrow in picture of day 30 depicts proliferating human hepatocytes with normal NTCP expression. The white arrows depict non-cycling human hepatocytes with weak or negative NTCP expression. Green spots seen in the pictures were non-specific. (C) Immunofluorescent staining of frozen sections for HBcAg (green), keratin 18 (red) and nuclei (blue). Keratin 18 was used to specifically recognize human hepatocytes in the mouse livers. Different days represent mice euthanized at different time points post second transplantation as indicated above. Scale bars = 50 μm.

To assess whether NTCP down-regulation contributed to the lack of susceptibility to HBV infection of proliferating human hepatocytes, we performed co-staining of NTCP, Ki67 and human hepatocyte marker in liver sections of mice that had received HBV-infected human hepatocytes and were euthanized at different time points post transplantation. As seen in Figure 2(B) and Supplemental Fig 2, when proliferative stimulus of human hepatocytes was strong at day 3 and day 14 (indicated by Ki67 and SP100 positive cells), NTCP protein level was sharply down-regulated or even undetectable. As human hepatocyte proliferation relented and most hepatocytes returned to the quiescent state (day 30 post-transplantation), NTCP protein level returned to normal and expressed persistently at the cell membrane. At the same time, serological viral markers began to rebound [10] and hepatocytes became HBcAg-positive at later time points (Figure 2(C) and [10]). Moreover, when treating the mice with the NTCP receptor inhibitor Myrcludex-B from day 30, the rebound of the HBV markers was blocked [10]. This data strongly indicated that down-regulation and low levels of membrane-associated NTCP render human proliferating hepatocytes poorly permissive or even resistant to HBV infection.

### Significantly lower NTCP expression is strongly correlated with more Ki67-positive hepatocytes and less HBsAg expression in HBV-related focal nodular hyperplasia (FNH) tissues

**Figure 3.**
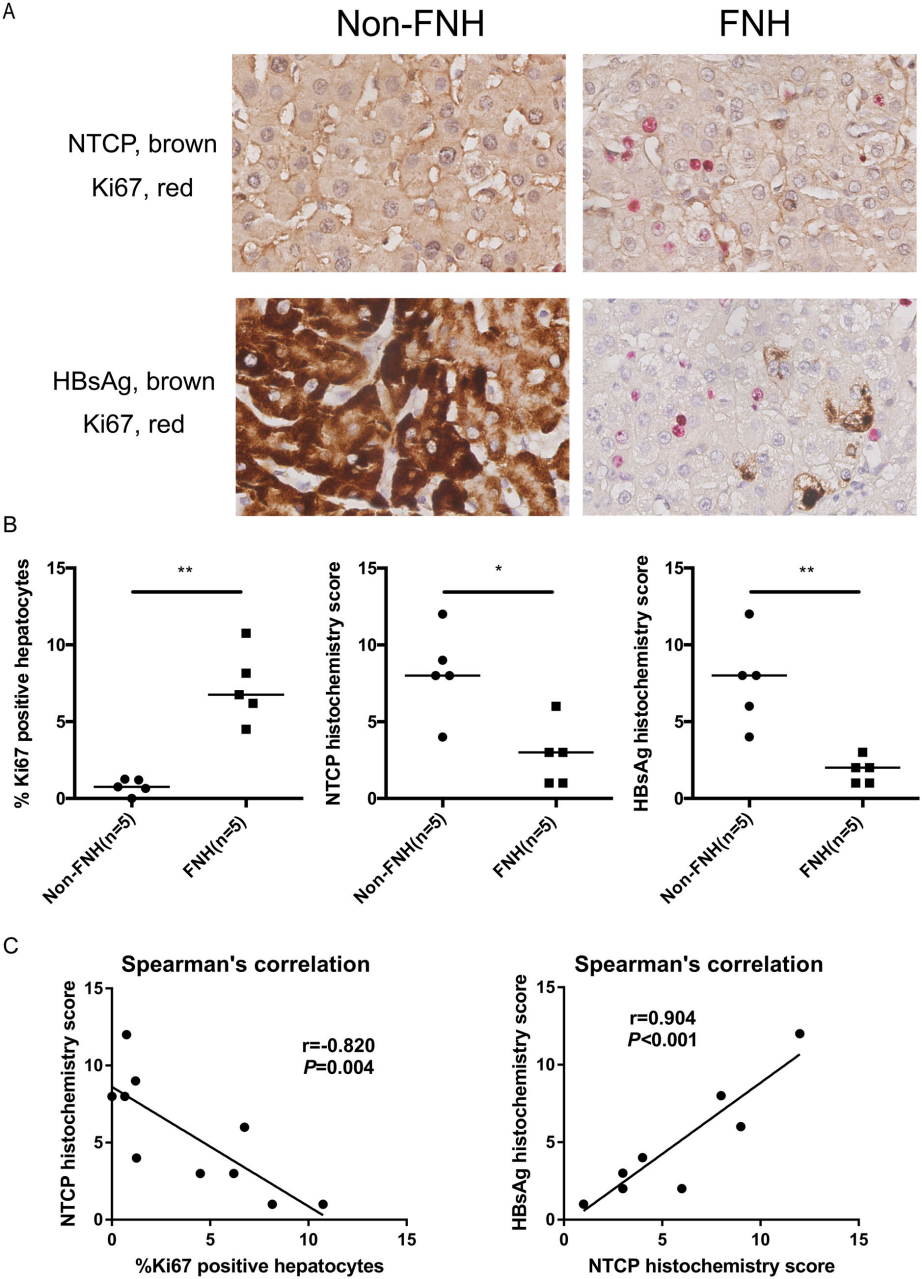
FNH tissues contain more Ki67-positive hepatocytes, along with significantly lower NTCP and HBsAg expression. (A) Immunohistochemical double staining of Ki67, NTCP and HBsAg in FNH tissues and adjacent non-FNH tissues. The top row showed the double staining of NTCP (brown) and Ki67 (red), and the bottom row showed the double staining of HBsAg (brown) and Ki67 (red). (B) The percentage of Ki67-positive hepatocytes (calculated by the average of five counted fields in every tissue) and the histochemistry scores of NTCP and HBsAg (calculated by positive hepatocyte ratio multiplied with the staining intensity). (C) Spearman's correlation of NTCP histochemistry scores and the percentage of Ki67-positive hepatocytes, the histochemistry scores of NTCP and HBsAg (*P < 0.05; **P < 0.01; ***P < 0.001).

The above data have demonstrated that NTCP down-regulation in proliferating hepatocytes is not conducive to HBV infection. FNH is the benign hepatocellular nodule of healthy liver, which is thought to occur as a result of a hyperplastic response of hepatocytes and bile ducts to a vascular anomaly [24]. For clinical validation, five pairs of HBV-related FNH tissues and adjacent non-FNH tissues were collected from surgical resection. As shown in Figure 3(A and B), compared to adjacent non-FNH tissues, more Ki67-positive hepatocytes were found in the FNH tissues. Meanwhile, expressions of NTCP and HBsAg were significantly lower in comparison to that in adjacent non-FNH tissues. Further, Spearman's correlation showed that NTCP histochemistry score was strongly negatively correlated with the percentage of Ki67-positive hepatocytes (r = −0.820, *P* = 0.004), and the histochemistry scores of NTCP and HBsAg were strongly positively correlated (r = 0.904, *P* < 0.001) (Figure 3(C)). These data implicate that proliferating hepatocytes may not favour HBV infection or viral antigen expression and that this phenomenon is strongly associated with NTCP down-regulation in the human liver.

### In CHB patients the down-regulation of NTCP expression is found correlated with higher inflammatory activity and more active hepatocyte compensatory proliferation

**Figure 4.**
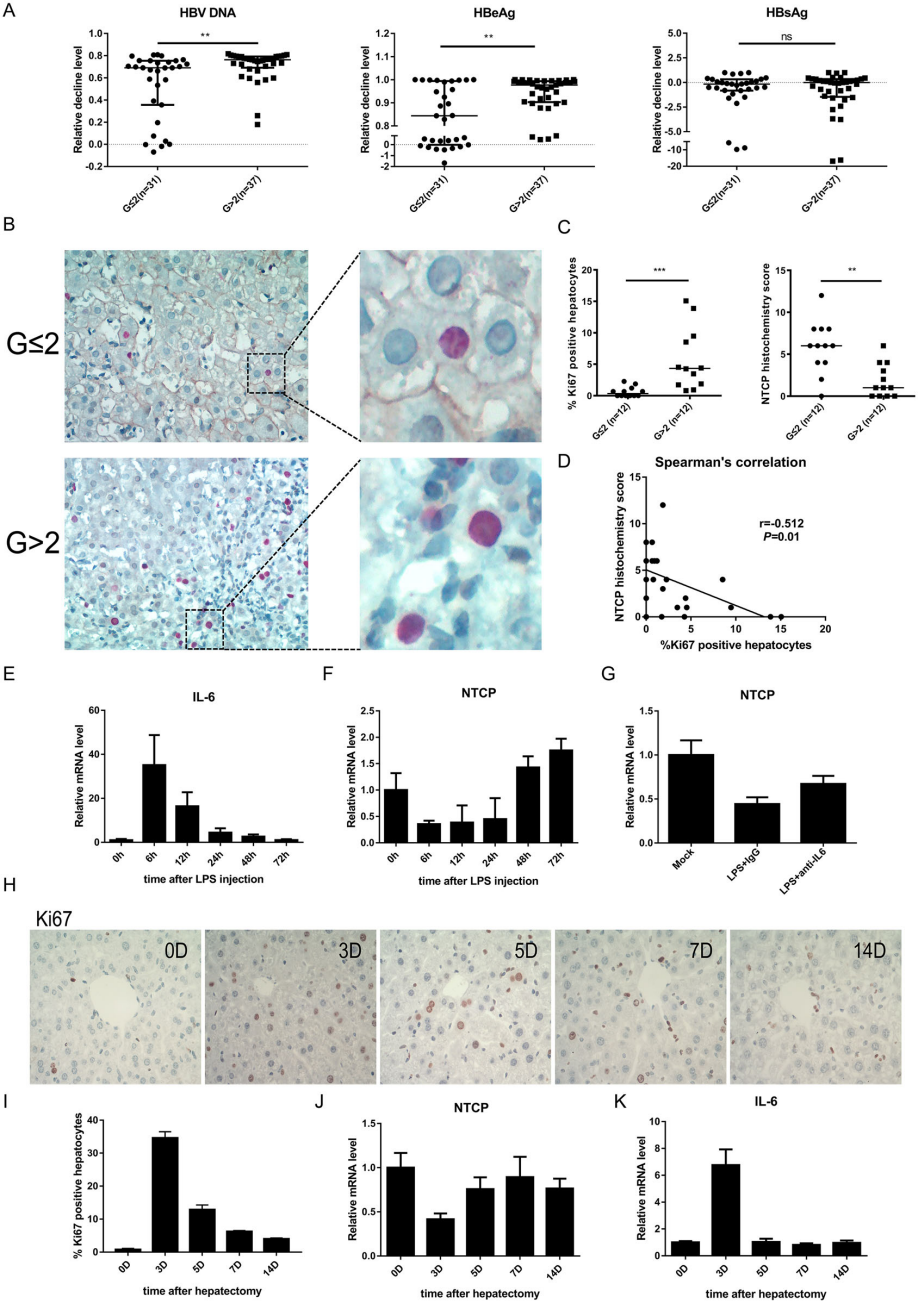
Down-regulation of NTCP in the liver of CHB patients with higher inflammatory activity and the probable regulatory mechanism. (A) Decline degrees of viral markers of CHB patients. Data calculated as the level before treatment minus the 6-month level after treatment and then divided by the level before treatment each patient. (B) Immunohistochemical double staining of NTCP (brown) and Ki67 (red) of liver biopsy specimens. (C) The percentage of Ki67-positive hepatocytes (calculated by the average of five counted fields each tissue) and NTCP histochemistry scores (calculated by positive hepatocyte ratio multiplying staining intensity). (D) Spearman's correlation of NTCP histochemistry scores and the percentage of Ki67-positive hepatocytes. (E) Real-time RT-PCR assay analysed mouse IL-6 mRNA expression after injection of LPS. (F and G) Real-time RT-PCR assay analysed mouse NTCP mRNA expression after injection of LPS (F) and after injection of LPS together with neutralizing serum IL-6 with IL-6 antibodies (G). (H) Ki67 immunohistochemical staining of mouse liver slices per time point after 70% partial hepatectomy. (I) The proliferation index was set up by counting the percentage of Ki67-positive hepatocytes on five fields per mouse and time point. (J and K) mRNA level of NTCP (J) and IL-6 (K) after mouse 70% partial hepatectomy at the indicated time points. Data were collected from three to five mice each time point. (**P < 0.01; ***P < 0.001; ns: not significant).

The results above have confirmed that the lower expression of cell membrane localized NTCP was unfavourable to HBV infection. To further explore the clinical significance, 68 HBeAg-positive chronic hepatitis B patients receiving either nucleos(t)ide analogues (NAs) and/or interferon therapy longer than 6 months were enrolled. According to their baseline (before antiviral treatment) necroinflammatory severities (G) evaluated by liver biopsy and pathologic diagnosis, these patients were divided into two groups: G ≤ 2 group and G > 2 group. As expected, the baseline ALT and AST levels in patients of G ≤ 2 group were significantly lower than that in G > 2 group (*P* < 0.0001, each), while no significant differences of the baseline virological parameters (HBsAg/HBeAg/HBV DNA) were found between the two groups (Table 1). As compared to those patients with liver inflammatory severity scored G2 or less, the G > 2 group had a significant decrease both in HBV DNA (*P* = 0.002) and HBeAg levels (*P* = 0.008) 6 months after the initiation of antiviral therapy (Figure 4(A)), indicating that the group with higher inflammatory activity had better virological response to antiviral therapy.

**Table 1.** Baseline characteristics of the patients enrolled (n=68), divided according to the inflammatory stage (G).

Variable	Total cohort	G ≤ 2	G > 2	*P*-value
	(n = 68)	(n = 31)	(n = 37)	
Gender, Male/Female	49/19	18/13	31/6	0.02
Age (years)	23.50 (15.75–33.5)	22.00 (12.00–36.00)	24.00 (5.00–32.00)	0.89
HBsAg (IU/mL)	3208.00 (1692.00–5185.00)	2057.00 (593.70–4429.00)	3852.00 (420.60–5319.00)	0.08
HBeAg (IU/mL)	927.25 (224.40–1195.00)	952.10 (5.20–1129.00)	686.70 (4.13–1312.00)	0.80
HBV DNA (Log10 IU/mL)	7.27 (6.13–8.00)	7.55 (3.84–8.05)	7.20 (3.99–8.00)	0.93
ALT(U/L)	185.50 (79.00–401.00)	93.00 (16.00–162.00)	354.00 (36.00–694.00)	<0.001
AST(U/L)	120.50 (61.5–245.0)	67.00 (24.00–103.00)	183.00 (37.00–373.00)	<0.001
Total bile acids (μmol/L)	11.00 (6.00–16.75)	10.00 (5.00–14.00)	13.00 (6.50–32.50)^#^	0.02

Data are expressed as median with range. *P-*values were calculated by Fisher’s exact test or non-parametric Mann-Whitney U test. *P-*value < 0.05(two-tailed) was considered to be statistically significant. ^#^: four patients with undetectable bile acids were excluded. For detail, 32 patients received mono NAs therapy, 5 patients received IFN only therapy, the remained 31 patients received NAs together with IFN.

Furthermore, 12 liver biopsy specimens from each group were collected for Ki67 and NTCP immunohistochemical double staining. As expected, the staining showed that the higher the inflammatory activity, the more the Ki67-positive cells, and the lower the NTCP expression. In general, a relative shallower NTCP staining was observed more often in Ki67-positive cells, as compared that with neighbouring cells (Figure 4(B, C)). Further, Spearman's correlation showed that NTCP histochemistry score was negatively correlated with Ki67-positive cell percentage (r = −0.512, *P* = 0.01) (Figure 4(D)). Interestingly, the bile acids level of G > 2 group was higher than that of G ≤ 2 group (Table 1). This phenomenon was in line with the physiological function of NTCP. The down-regulation of NTCP in G > 2 group resulted in less bile acid transport from blood to hepatocytes and high level of blood bile acids was observed.

Apart from the low NTCP protein level in proliferating hepatocytes, it was worth noticing that the overall NTCP level was lower in G > 2 group (Figure 4(B)), suggesting that inflammation-related cytokines may also down-regulate NTCP. Interleukin-6 **(**IL-6) is an important cytokine involved in inflammation and liver regeneration. It has been reported that high levels of the serum Interleukin-6 **(**IL-6) are found in CHB patients [25,26], and IL-6 can inhibit the expression of NTCP in primary human hepatocytes and in a differentiated cell line [16]. Therefore, the lipopolysaccharide (LPS) induced mouse model was used to mimic the inflammatory process to some extent. As expected, a sharp rise of IL-6 mRNA level was observed after injection of LPS (Figure 4(E)) and NTCP levels showed an inverse trend to that of IL-6 (Figure 4(F)). After neutralizing serum IL-6 with IL-6 antibodies, NTCP expression had some certain level of recovery (Figure 4(G)). In concordance, after mouse 70% partial hepatectomy, proliferative stimulus was strongest at day 3 (mean 35% Ki67-positive hepatocytes) (Figure 4(H, I)), while NTCP mRNA levels decreased (Figure 4(J)). On the contrary, IL-6 mRNA levels increased at day 3, when the same mice were analysed (Figure 4(K)). These results suggest that IL-6 could inhibit the expression of NTCP both in inflammation and liver regeneration.

Taken together, the histological result suggested that NTCP is down-regulated in the liver of CHB patients with higher inflammatory activity and more active hepatocyte compensatory proliferation. Since the above data showed that down-regulation of NTCP in proliferating hepatocytes leads to HBV infection resistance, we speculated that the low level of NTCP caused nascent hepatocytes to resist HBV infection, which might facilitate CHB patients with higher inflammatory activity and more active hepatocyte compensatory proliferation to achieve efficient virological response during antiviral therapy.

### Cell cycle regulating genes p53 and SKP2 transcriptionally down-regulate NTCP expression

**Figure 5.**
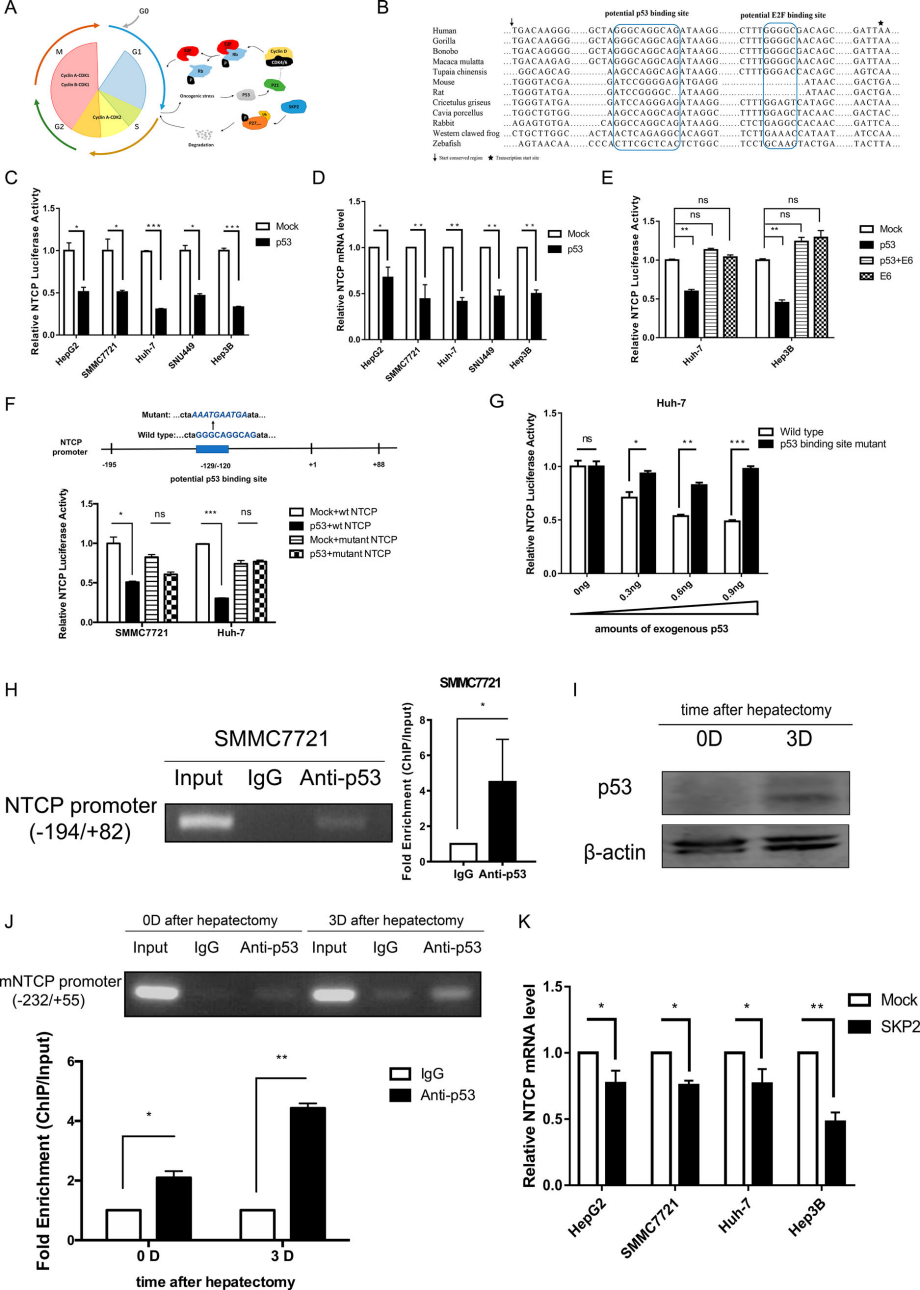
p53 and SKP2 transcriptionally inhibit NTCP expression. (A) Schematic diagram of cell cycle regulation. (B) The potential p53 binding site and potential E2F binding site in the highly conserved NTCP promoter region. (C, E) Luciferase reporter assays were conducted to analyse the activity of NTCP promoter in HCC cells which were transfected with p53 or p53 and HPV E6 plasmids. (D) Real-time RT-PCR analysis of p53 inhibiting NTCP mRNA level in HCC cells. (F) Luciferase reporter assays were conducted to analyse the effect of p53 on the activity of NTCP promoter containing the mutant p53 binding site. (G) Luciferase reporter assays were conducted to analyse the effect of different amounts of p53 plasmids (as shown in the X-axis) on the activity of NTCP promoter or NTCP promoter containing the mutant potential p53 binding site. (H, J) ChIP-PCR assays were conducted to determine the binding of p53 on NTCP promoter in SMMC7721 cells (H) and mouse liver tissues (J). (I) The p53 protein level of mouse liver in day 0 and day 3 after hepatectomy. (K) The inhibition of SKP2 on NTCP mRNA level. (*P < 0.05; **P < 0.01; ***P < 0.001; ns: not significant).

Since NTCP is down-regulated during hepatocyte proliferation, we speculate that the down-regulation of NTCP is associated with cell cycle regulatory genes. When cells are stimulated by mitogen-growth factors, cyclin D1 continuously accumulates, associates with and activates CDK4/6. The activated cyclin D1/CDK4/6 complexes then phosphorylate retinoblastoma protein (Rb), which leads to transcription factor E2F release and promotes transcription of many genes associated with cell cycle progression [27]. At this time, the p53 pathway is activated to prevent any potential malignant proliferation of the cells (Figure 5(A)) [28]. Interestingly, bioinformatics analysis identified a potential p53 binding site in the highly conserved promoter sequence of NTCP (Figure 5(B)). To test whether p53 down-regulates NTCP, a plasmid containing the highly conserved promoter region of NTCP was constructed, and the luciferase reporter assays showed that exogenous p53 expression caused a 50–75% reduction to NTCP promoter activity in all five cell lines tested (Figure 5(C)). In line with this, the endogenous NTCP mRNA level in these cell lines was also down-regulated (Figure 5(D)). Moreover, the p53-mediated inhibition of NTCP promoter activity was itself inhibited by co-expression of HPV E6 gene, a viral protein which is known to promote the poly-ubiquitination of p53 and lead to its degradation (Figure 5(E)) [29]. When the potential p53 binding site in the NTCP promoter was mutated, exogenously expressed p53 no longer inhibited NTCP promoter activity (Figure 5(F)). Consistent with these data, a p53 dosage-dependent inhibition was exhibited on the transcriptional activity of wild-type NTCP promoter, but not on that carrying a mutant p53 binding-site (Figure 5(G)). To further demonstrate the interaction of p53 with the predicted binding site on NTCP promoter, ChIP combined with PCR assays were performed in SMMC7721 cells (containing wild-type p53) transfected with a p53 expression plasmid. As shown in Figure 5(H), PCR of the NTCP promoter using the DNA fragment immunoprecipitated by the p53 antibody showed that p53 was able to specifically bind to the highly conserved NTCP promoter (left panel), quantification of this result using real-time PCR demonstrated a greater than five-fold increase in the NTCP promoter region binding to p53 (right panel). After mouse hepatectomy, NTCP mRNA level significantly decreased at day 3 (Figure 4(J)), while p53 protein level raised (Figure 5(I)). In line with these observations, ChIP-PCR assays showed that p53 bound to the mouse NTCP promoter at day 0, and that the binding was stronger at day 3 after hepatectomy (Figure 5(J), top panel). Quantification of the amounts NTCP promoter in DNA fragment using real-time PCR further confirmed the results (Figure 5(J), bottom panel). Taken together, these results suggest that p53 is able to specifically bind to the highly conserved NTCP promoter region and down-regulate NTCP mRNA level during hepatocyte proliferation.

S-phase kinase-associated protein 2 (SKP2) can ubiquitinate and degrade many cell cycle-related proteins such as p27 and promote cell cycle progression from G1 to S (Figure 5(A)) [30]. Accordingly, we found that exogenous expression of SKP2 could slightly inhibit the mRNA expression level of NTCP in HepG2, SMMC7721 and Huh-7 cell lines. Nevertheless, such inhibition exacerbated to a 50% decrease in the p53-null Hep3B cell line (Figure 5(K)). It has been reported that SKP2 could down-regulate p53 protein level in colorectal carcinoma cell lines or up-regulate p53 protein level in human melanoma cell lines [31,32]. However, in our preliminary study, it was seen that overexpression of SKP2 had no effect on p53 protein in HCC cell lines (Supplemental Fig 3). These results indicate that the inhibition of SKP2 on NTCP is not dependent on p53 and should be studied further in the future.

**Figure 6.**
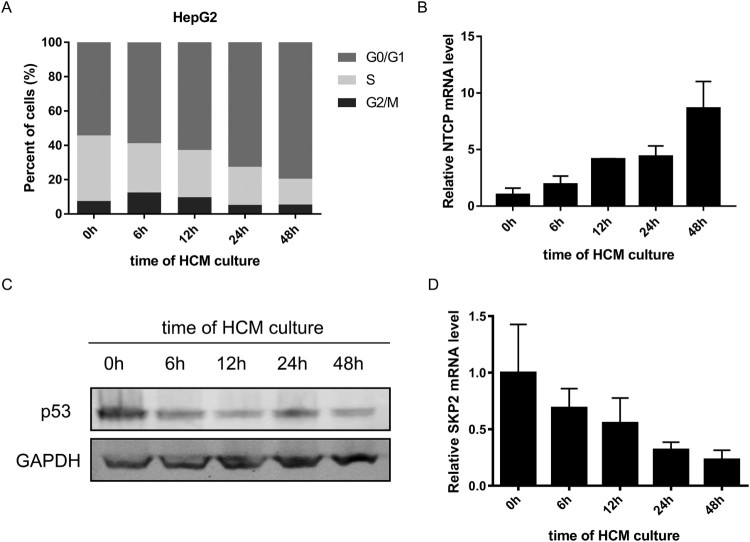
Changes of NTCP, p53 and SKP2 during proliferation/ quiescence translation of HepG2 cells. (A) Changes of the percentage of HepG2 cells in each phases of cell cycle (tested by flow cytometry cell cycle assays) at the different time points of HCM culturing. (B-D) changes of NTCP mRNA level (B), p53 protein level (C), SKP2 mRNA level (D) in HepG2 cells during culturing in HCM for different time points.

In order to explore whether p53 and SKP2 transcriptionally down-regulating NTCP expression happened in cell cycle translation, HepG2 cells were progressively arrested in the G0/G1 phase by culturing in HCM for different time points, as tested by flow cytometry cell cycle assays (Figure 6(A)). Meanwhile, the mRNA level of NTCP gradually increased (Figure 6(B)). In contrast, the mRNA level of SKP2 and the protein level of p53 gradually decreased (Figure 6(C, D)). Taken together with the above experiments, these results prompt that p53 and SKP2 could down-regulate NTCP expression during the quiescence/proliferation translation phase of hepatocytes.

### Cyclin D1 inhibiting NTCP promoter activity is p53 dependent

**Figure 7.**
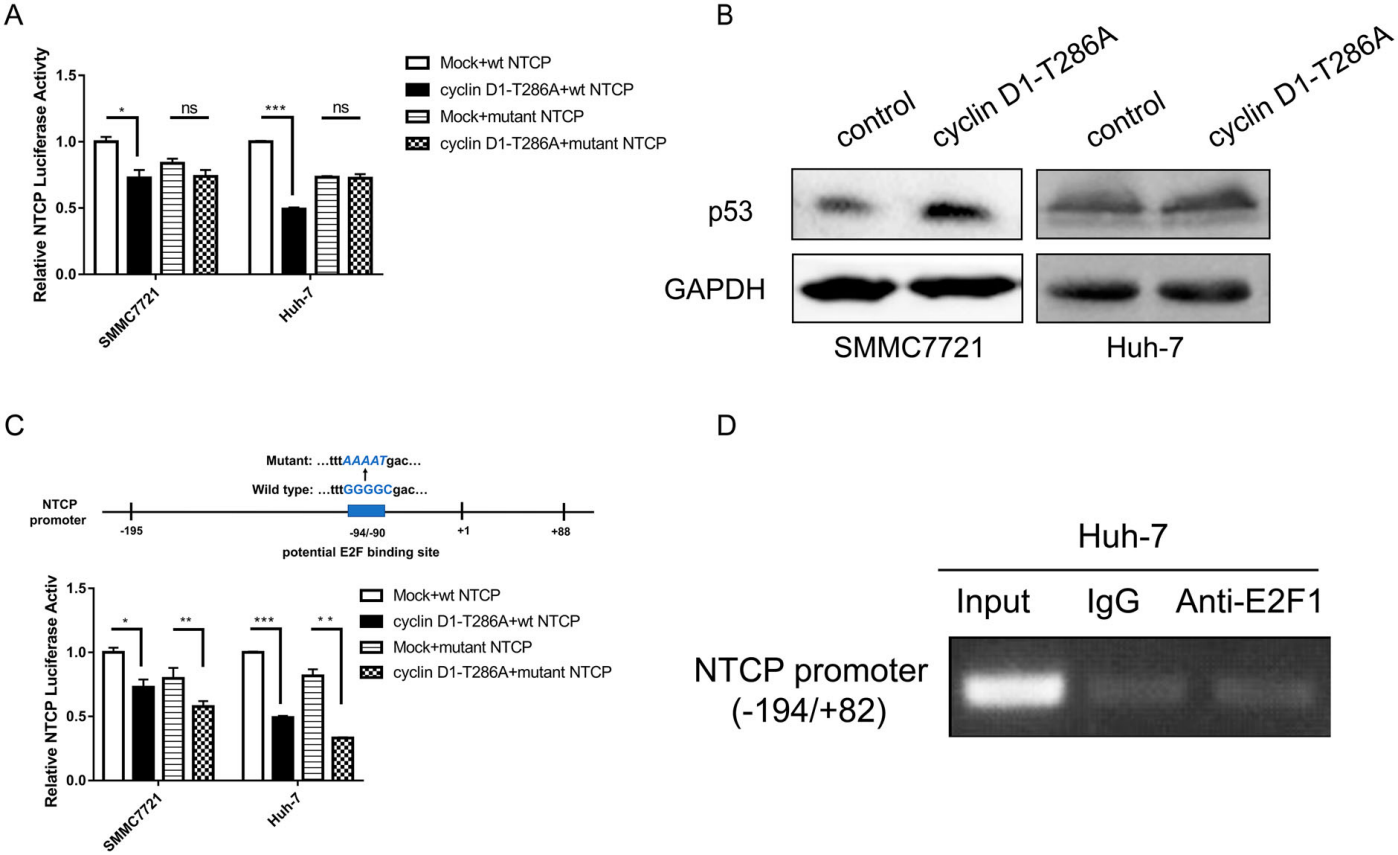
Cyclin D1 transcriptional inhibiting NTCP is p53 dependent. (A) NTCP promoter activity was tested by dual luciferase reporter system. Cyclin D1-T286A plasmid was co-transfected with wild type NTCP promoter or NTCP promoter containing the mutant p53 binding site (Figure 5(F)). (B) The protein level of p53 in SMMC7721 and Huh-7 after transfected with cyclinD1-T286A plasmid were detected by western blot. (C) Luciferase reporter assays were conducted to analyse the effect of cyclin D1-T286A on the activity of NTCP promoter containing the mutant E2F binding site. (D) ChIP-PCR assay of E2F1 not binding to the highly conserved NTCP promoter in Huh-7 cells. (*P < 0.05; **P < 0.01; ***P < 0.001; ns: not significant).

Our previous result has shown that ectopic expression of the constant active form of cyclin D1 (T286A mutant) [33] caused a significant decrease of the NTCP promoter activity in the HCC cell lines SMMC7721 containing wild-type p53 and Huh-7 bearing a p53-Y220C mutation [20]. Surprisingly, when the p53 binding site was mutated, ectopic expression of cyclin D1-T286A mutant no longer suppressed the activity of NTCP promoter ([20], Figure 7(A)). More supporting data was obtained that exogenous expression of cyclin D1-T286A could up-regulate the endogenous p53 protein level (Figure 7(B)). Though a potential E2F binding site was also found in the NTCP promoter region (Figure 5(B)), the T286A mutant of cyclin D1 still suppressed NTCP promoter activity with the mutant potential E2F binding site (Figure 7(C)). Further ChIP-PCR assays also showed that E2F1 did not bind to the highly conserved NTCP promoter region (Figure 7(D)). Taken together these results demonstrate that the inhibitory effect of cyclin D1 on NTCP promoter activity may depend on p53 rather than E2F.

## Discussion

NTCP is the main hepatocellular sodium-dependent transporter for conjugated bile acids in human and rodent livers, which has also been identified as the functional receptor for HBV in human hepatocytes [34–36]. It is known that HBV infection can be blocked by NTCP-inhibiting drugs and substrates, providing direct evidences that NTCP expression level or its binding capacity to HBV affects infection efficiency [37]. In addition, Miura et al. found that liver regeneration could change the sub-cellular localization of NTCP from basolateral membrane to the cytoplasm of hepatocyte [19]. In line with this, when DOX-treated HepG2-NTCP-tet cells were arrested in G0/G1 phase, the cell membrane expression of NTCP was indeed increased and NTCP located in the cytoplasm turned onto cell membrane. As a consequence, HBV infection efficacy rose. It has been reported that NTCP protein could be degraded by the ubiquitin-proteasome system and by the lysosome system, among which the latter one seems to play a major role [23,38,39]. In this study, we demonstrated that lysosomal-mediated degradation of NTCP protein could be inhibited when HepG2-NTCP cells were arrested in G0/G1 phase. Thus, the enhanced NTCP degradation determined in proliferating hepatocytes might in part explain the lower levels of NTCP protein detected in proliferating hepatocytes. In addition, it seems likely that hepatocyte proliferation not only decreases the levels of NTCP, but also changes its cell membrane localization and thereby inhibits its function as an HBV entry receptor. It has been reported that cAMP could regulate the sub-cellular localization of NTCP protein through several pathways, including PI3K/Akt, PI3K/PKCδ, PI3K/PKCζ, and PP2B [40,41]. Whether these pathways participate in the trafficking of NTCP protein during the quiescence/proliferation translation of hepatocytes remains to be explored. Further in vivo experiments performed using HBV-infected liver-humanized mice demonstrated that in human hepatocytes, the protein levels and cell membrane localization of NTCP recovered to normal only when liver regeneration relented or was almost accomplished. Concordantly, HBV reinfection only happened thereafter [10]. During the early phases of liver repopulation, the level of NTCP protein expressed in human hepatocytes was low in general. Interestingly, the expression of NTCP protein in some Ki67-negative human hepatocytes was also low. Moreover, while NTCP protein level returned to normal in most hepatocytes at later time points (day 30 after transplantation), we observed both proliferating human hepatocytes with normal NTCP protein level (blue arrow in Figure 2(B)), as well as non-cycling hepatocytes with weak or even negative NTCP expression (white arrows in Figure 2(B)). This strong but transient change of NTCP expression and localization resembles alterations that are also observed in de-differentiated hepatocytes and in hepatoma cell lines. Thus, our results show that not only cell de-differentiation but also the proliferation of human hepatocytes in the setting of liver regeneration is sufficient to promote the down-regulation of NTCP protein. According to this scenario, proliferating human hepatocytes appear less susceptible to HBV infection.

It has been suggested that in patients with acute or chronic hepatitis B, CD8+ T cells could directly kill HBV-infected cells, which induces proliferation of neighbouring HBV-infected and uninfected hepatocytes to compensate for cell loss [42]. The cccDNA in the nuclei of infected hepatocytes will be diluted or even be depleted during the hepatocyte compensatory proliferation [10]. Meanwhile, accumulating evidences have shown that inflammatory cytokines like IFN-γ and TNF-α secreted from T cells could control and even eliminate HBV, through inhibiting HBV replication, eliminating capsids and by destabilizing cccDNA [43]. Also, liver-enriched transcriptional factors such as HNF-4α, which could support HBV transcription and replication, are down-regulated in this process [44]. All these mechanisms could contribute to the virus clearance of HBV infection. However, the mechanism relevant to how the proliferating hepatocytes being prevented from HBV reinfection remained as an unsolved puzzle. Based on the discoveries in this study, we proposed that the down-regulation of cell membrane localized expression of NTCP protein contributes to the resistance of HBV infection seen in those proliferating hepatocytes. Additionally, we provided histological evidence that NTCP is significantly down-regulated in CHB patients with higher inflammatory activity and more proliferating hepatocytes, who are also likely to achieve better antiviral therapy efficacy. Taken together, this might be a timely reminder that NTCP down-regulation mediated resistance of HBV infection in nascent proliferating hepatocytes play a synergistic role to the virus clearance of acute HBV infection and the efficient virological response to antiviral therapy in CHB patients with moderate or severe liver necroinflammation.

It has been estimated that after hepatectomy, reconstitution of the entire liver mass is complete within 5–7 days in rodents. Furthermore, virtually all remaining hepatocytes divide once or twice to restore the original liver mass within 3–4 days [45]. In line with these observations, we found that after hepatectomy the mRNA levels of mouse NTCP were down-regulated at day 3 and gradually recovered from day 5. Further in vitro experiments showed that NTCP levels increased significantly when the proliferating HepG2 cells were forced into G0/G1 phase by HCM. Both the in vivo and in vitro results provide direct evidences that NTCP is transcriptionally down-regulated in proliferating hepatocytes. Our previous study showed that cyclin D1 could down-regulate NTCP expression in HBV-related hepatocellular carcinoma [20]. In this study, using several hepatoma cell lines we uncovered for the first time that p53 is the dominant transcription regulator in proliferating hepatocytes responsible for the down-regulation of NTCP expression, which was supported by the observation that p53 protein could bind to NTCP promoter region in mouse liver tissues. Furthermore, cyclin D1 exerts its function by transcriptionally inhibiting NTCP expression via p53. Previous studies demonstrated that p53 could suppress HBV replication by binding and repressing the HBV enhancer [46]. Here, we provide an alternative mechanism by which p53 could inhibit the entry of HBV into hepatocytes by transcriptionally down-regulating NTCP. In this study, we also found that SKP2 may compensate for the effect of p53 in inhibiting NTCP expression, since exogenous overexpression of cell cycle positive regulatory gene SKP2 could significantly decrease the mRNA level of NTCP in the p53-null Hep3B cell line, while only slightly down-regulation of NTCP was observed in HepG2, SMMC7721 and Huh-7 cell lines.

In conclusion, the current study demonstrated that the level of cell membrane localized NTCP is suppressed in proliferating hepatocytes, which is likely to play a major role in determining resistance to HBV infection. Clinically, in consideration of the uncountable cycles of hepatocyte death and regeneration during acute HBV infection and CHB with persistent inflammation, we suggest that NTCP down-regulation in nascent proliferating hepatocytes might also contribute to the control or elimination of the virus. Mechanistically, it was proved that cell cycle regulating genes p53, cyclin D1 and SKP2 could transcriptionally down-regulate NTCP expression in proliferating hepatocytes.

## Declarations

**Ethics approval of animals:** The animal experiments involving humanized mice were approved by the City of Hamburg, Germany (G118/16), conducted in accordance with the European Communities Council Directive (86/EEC). The other animals involved in the study were approved by the Bioethics Committees of Peking University, the reference number is LA2018181. All experimental researches on animals followed internationally recognized guidelines.

**Ethics approval and consent to participate:** The clinical data involved in the study was approved by the Bioethics Committees of Peking University at February 27th, 2018. We confirm that we had all necessary consent from any patients involved in the study, including consent to participate.

**Availability of data and materials:** The datasets used during the current study are available from the corresponding author on reasonable request.

**Author contributions**: These authors were involved with this manuscript: Fengmin Lu, Maura Dandri, Jingmin Zhao and Xiangmei Chen (study concept and design, analysis and interpretation of data); Fengmin Lu, Ying Yan, Danli Yang and Lena Allweiss (drafting of the manuscript); Ying Yan, Lena Allweiss, Danli Yang, Jingting Kang, Jianwen Wang, Xiangjun Qian, Ting Zhang, Hui Liu, Lu Wang, and Shuhong Liu (acquisition of data; analysis and interpretation of data; statistical analysis); Jianhua Sui (technical or material support).

## Supplementary Material

Supplemental Material
