# Supplementary material for: Down-regulation of cell membrane localized NTCP expression in proliferating hepatocytes prevents hepatitis B virus infection
Source: Emerg Microbes Infect. 2019 Jun 9;8(1):879–94. doi: 10.1080/22221751.2019.1625728 (PMC6567113; doi:10.1080/22221751.2019.1625728)
Supplement: Supplemental Material [file TEMI_A_1625728_SM4855.zip › 10. Supplementary (new).docx]

**SUPPLEMENTARY**

**
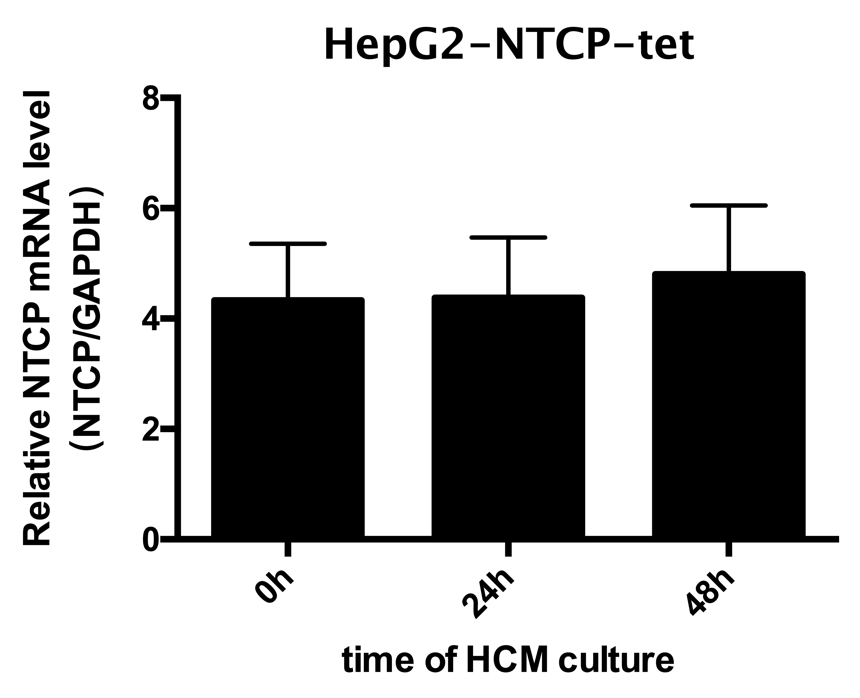
**

**Supplemental Figure 1.** **Changes of NTCP mRNA level** **when DOX-treated HepG2-NTCP-tet cells were cultured in HCM for 24h or 48h.**

**
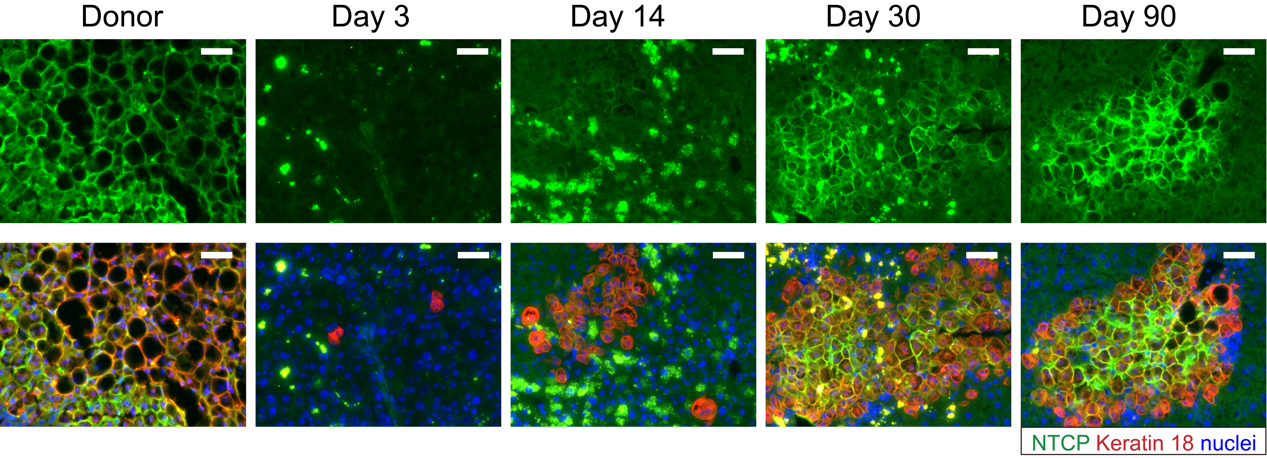
**

**Supplemental Figure 2. Immunofluorescent staining of NTCP (green), keratin 18 (red) and nuclei (blue) in the liver tissues of USB mice.** Keratin 18 was used to specifically recognize human hepatocytes in the mouse livers. Donor represents donor mice. Different days represent mice euthanized at different time points post second transplantation. The green spots seen in the pictures were non-specific.


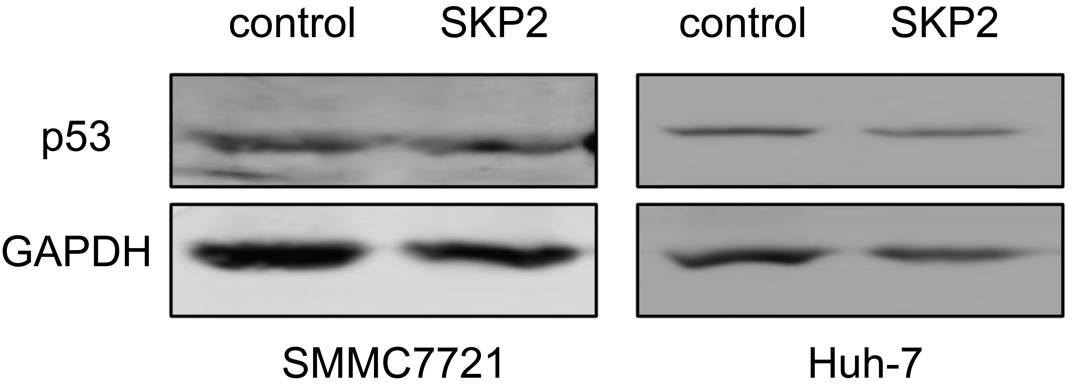


**Supplemental Figure 3. Ectopic overexpression of SKP2 in HCC cell lines had no effect on P53 protein level.**

**Supplemental Table 1**

The primer sequences used for Real-time RT-PCR

| **Gene name** | **Forward (5’-3’)** | **Reverse (5’-3’)** |
| --- | --- | --- |
| hNTCP | TGACCACCTGCTCCACCTTC | GAATGAGAACCAGGACCAGTGAT |
| hSKP2 | TTTCATGGGACTCCCTTCCG | GAGACAGTATGCCGTGGAGG |
| hGAPDH | ACCACAGTCCATGCCATCAC | TCCACCACCCTGTTGCTGTA |
| mNTCP | ATCATGCTCTCGCTTGGCTG | AGGATGGCCAGAGCCTCAAT |
| mIL-6 | GCTTAATTACACATGTTCTCT  GGGAAA | CAAGTGCATCATCGTTGTTCATAC |
| mGAPDH | GGTGAAGGTCGGTGTGAACG | CTCGCTCCTGGAAGATGGTG |

**Supplemental Table 2**

The primer sequences used to construct NTCP luciferase reporter vector and binding site mutations.

| **The primer sequences used for WT NTCP promoter clone** | | |
| --- | --- | --- |
| pGL3-NTCP | Forward primer | CCTCGAGGTGACAAGGGAGGAGTACAAGTAGCACCCAG |
|  | Reverse primer | CAAGCTTGCTCCATCCTCCTGTGAGGCAGTGGAAGACCACTC |
| **The primer sequences used for mutant NTCP promoter clone** | | |
| pGL3-NTCP promoter-P53 binding site mutant | Forward primer | CAGGGAGCTTGGGAGAGCTAAAATGAATGAATAAGGTTCTGGGCTGTTCC |
|  | Reverse primer | GGAACAGCCCAGAACCTTATTCATTCATTTTAGCTCTCCCAAGCTCCCTG |
| pGL3-NTCP promoter-E2F binding site mutant | Forward primer | GTTCTGGGCTGTTCCTCTTTAAAATGACAGCCAGAGAAATAGCTC |
|  | Reverse primer | GAGCTATTTCTCTGGCTGTCATTTTAAAGAGGAACAGCCCAGAAC |
