# Supplementary figures and images for: Down-regulation of cell membrane localized NTCP expression in proliferating hepatocytes prevents hepatitis B virus infection
Source: Emerg Microbes Infect. 2019 Jun 9;8(1):879–94. doi: 10.1080/22221751.2019.1625728 (PMC6567113; doi:10.1080/22221751.2019.1625728)

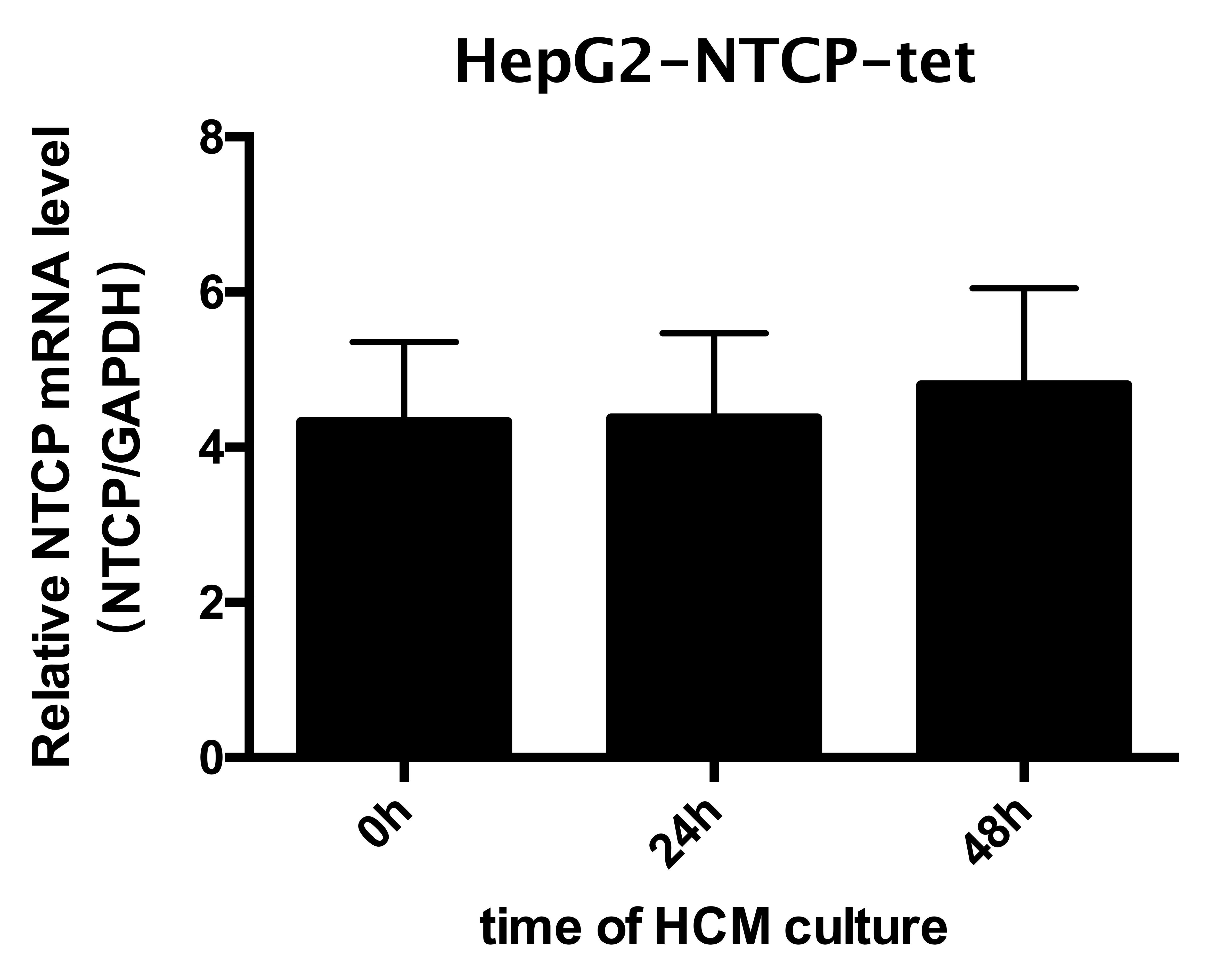

Supplement: Supplemental Material [file TEMI_A_1625728_SM4855.zip › 11. Supplemental Figure 1 (new).png]

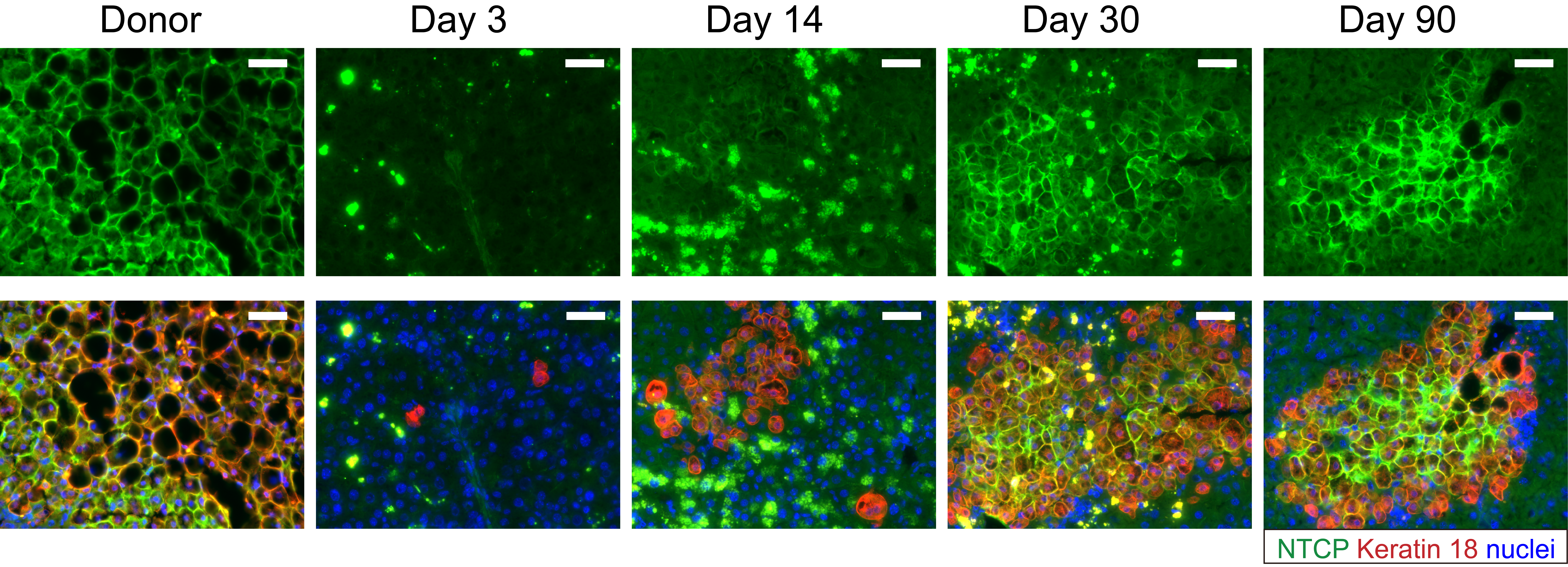

Supplement: Supplemental Material [file TEMI_A_1625728_SM4855.zip › 12. Supplemental Figure 2(new).png]

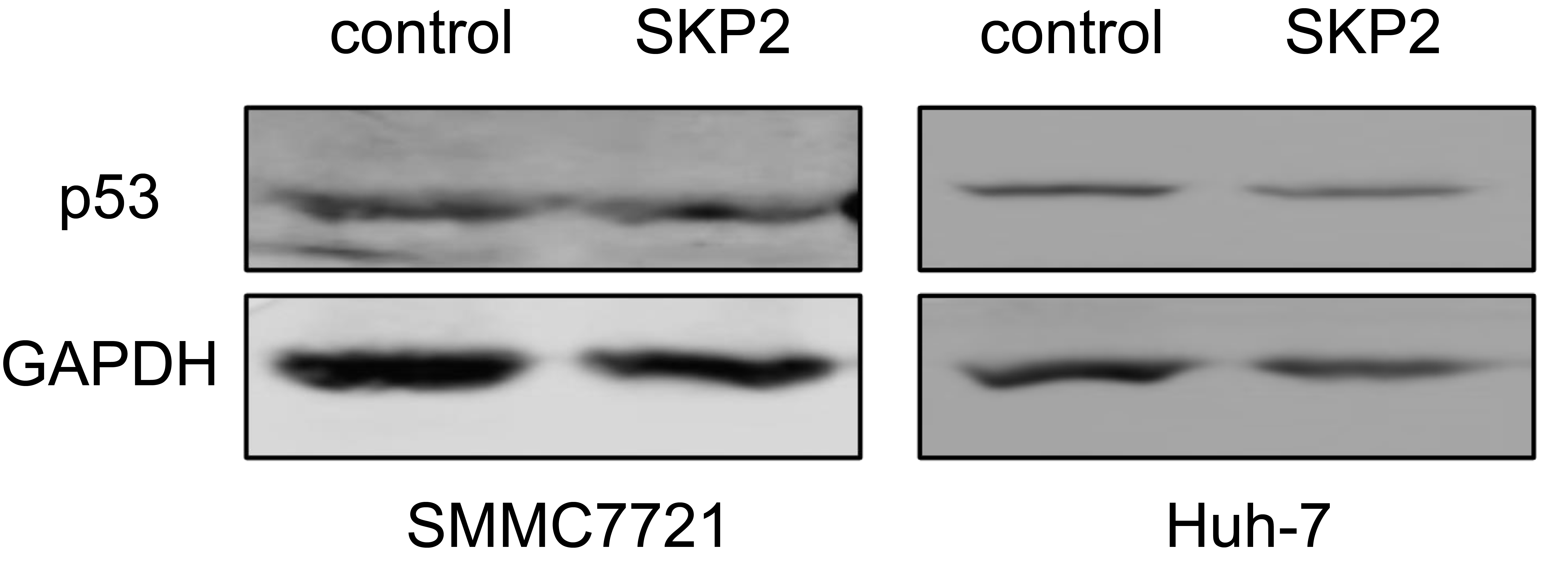

Supplement: Supplemental Material [file TEMI_A_1625728_SM4855.zip › 13. Supplemental Figure 3(new).png]
